# Texture Analysis of Fractional Water Content Images Acquired during PET/MRI: Initial Evidence for an Association with Total Lesion Glycolysis, Survival and Gene Mutation Profile in Primary Colorectal Cancer

**DOI:** 10.3390/cancers13112715

**Published:** 2021-05-31

**Authors:** Balaji Ganeshan, Kenneth Miles, Asim Afaq, Shonit Punwani, Manuel Rodriguez, Simon Wan, Darren Walls, Luke Hoy, Saif Khan, Raymond Endozo, Robert Shortman, John Hoath, Aman Bhargava, Matthew Hanson, Daren Francis, Tan Arulampalam, Sanjay Dindyal, Shih-Hsin Chen, Tony Ng, Ashley Groves

**Affiliations:** 1 Research Department of Imaging, Division of Medicine, University College London (UCL), London WC1E 6BT, UK; kenneth.miles@ucl.ac.uk (K.M.); s.punwani@ucl.ac.uk (S.P.); d.walls@ucl.ac.uk (D.W.); l.hoy@ucl.ac.uk (L.H.); john.hoath@nhs.net (J.H.); yevgenyc@cgmh.org.tw (S.-H.C.); ashleygroves@nhs.net (A.G.); 2 Imaging Division, Surgery and Cancer Board, University College London Hospitals (UCLH) NHS Foundation Trust, University College Hospital (UCH), London NW1 2BU, UK; a.afaq@nhs.net (A.A.); manuel.rodriguez@nhs.net (M.R.); mwan@nhs.net (S.W.); saif.khan4@nhs.net (S.K.); raymond.endozo@nhs.net (R.E.); robertshortman@nhs.net (R.S.); sanjay.dindyal@nhs.net (S.D.); 3 Department of Radiology, Carver College of Medicine, University of Iowa, Iowa City, IA 52242, USA; 4 Institute of Health Barts and London Medical School, Queen Mary University of London (QMUL), London E1 2AD, UK; amanbhargava@nhs.net; 5 Division of Cancer and Clinical Support, Barking, Havering and Redbridge University Hospitals NHS Trust, Queens and King George Hospitals, Essex IG3 8YB, UK; mhanson1@nhs.net; 6 Department of Colorectal Surgery, Royal Free London NHS Foundation Trust, Barnet and Chase Farm Hospitals, London NW3 2QG, UK; daren.francis@nhs.net; 7 Department of Surgery, East Suffolk and North Essex NHS Foundation Trust, Colchester General Hospital, Colchester CO4 5JL, UK; t.arulampalam@nhs.net; 8 Department of Nuclear Medicine, Keelung Chang Gung Memorial Hospital, Keelung 204, Taiwan; 9 School of Cancer & Pharmaceutical Sciences, King’s College London (KCL), London WC2R 2LS, UK; tony.ng@kcl.ac.uk

**Keywords:** colorectal cancer, magnetic resonance imaging, Dixon sequence, positron emission tomography, texture analysis

## Abstract

**Simple Summary:**

There is a need to demonstrate additional clinical value/utility for PET/MRI in oncology which moves beyond simple diagnosis. We believe that our currently submitted work represents an initial step in realizing the goal of moving PET/MRI beyond simple diagnosis. We report how texture analysis of parametric images depicting tumor fractional water content derived from routine PET/MRI Dixon acquisitions shows good inter-operator agreement, generates biologically relevant information related to total lesion glycolysis and gene mutation count, and provides prognostic information that can potentially unlock new clinical applications for patients with colorectal cancer. This research study resulted from a long-standing multi-disciplinary collaboration between the nuclear medicine physicians at our institute and experts in quantitative imaging, MRI radiology, GI histology, clinical oncology in colorectal cancer, and several gastrointestinal surgeons at various local hospitals.

**Abstract:**

To assess the capability of fractional water content (FWC) texture analysis (TA) to generate biologically relevant information from routine PET/MRI acquisitions for colorectal cancer (CRC) patients. Thirty consecutive primary CRC patients (mean age 63.9, range 42–83 years) prospectively underwent FDG-PET/MRI. FWC tumor parametric images generated from Dixon MR sequences underwent TA using commercially available research software (TexRAD). Data analysis comprised (1) identification of functional imaging correlates for texture features (TF) with low inter-observer variability (intraclass correlation coefficient: ICC > 0.75), (2) evaluation of prognostic performance for FWC-TF, and (3) correlation of prognostic imaging signatures with gene mutation (GM) profile. Of 32 FWC-TF with ICC > 0.75, 18 correlated with total lesion glycolysis (TLG, highest: r_s_ = −0.547, *p* = 0.002). Using optimized cut-off values, five MR FWC-TF identified a good prognostic group with zero mortality (lowest: *p* = 0.017). For the most statistically significant prognostic marker, favorable prognosis was significantly associated with a higher number of GM per patient (medians: 7 vs. 1.5, *p* = 0.009). FWC-TA derived from routine PET/MRI Dixon acquisitions shows good inter-operator agreement, generates biological relevant information related to TLG, GM count, and provides prognostic information that can unlock new clinical applications for CRC patients.

## 1. Introduction

By combining two powerful imaging modalities, PET/MRI has the potential to significantly impact the care of patients with cancer. However, current research has shown only limited diagnostic benefit over PET/CT and supplementary clinical roles need to be identified to support the use of PET/MRI in routine clinical practice [1]. Extending the range of biologically relevant information generated from PET/MRI by exploiting the quantitative imaging capabilities of the MRI component represents one approach to achieving this goal. Discovery of novel parameters with prognostic or predictive significance could create opportunities for PET/MRI to contribute to precision medicine [1].

Attenuation correction (AC) of PET images acquired using oncological PET/MRI is commonly performed using two-point Dixon sequences which separate fat and water tissue components. However, the biological relevance and possible clinical value of quantitative analysis of these sequences during oncological PET/MRI remains relatively unexplored. A single study incorporated quantitative analysis of separate fat- and water- weighted images derived from two-point Dixon sequences into a PET/MRI radiomic study of renal cancer but did not use the data to generate parametric maps of absolute fractional fat or water content within the tumor [2]. To date, PET/MRI measurements of the fractional fat (signal fat fraction) or water content of tissues have been limited to comparative assessments of bone marrow factional water content and glucose metabolism in healthy volunteers [3], a cohort of oncological patients with a variety of tumor types [4], and a series of patients with plasma cell dyscrasias [5]. The authors have been unable to identify any PET/MRI studies reporting quantitative imaging of fractional water content in solid tumors, despite previous pre-clinical research indicating increased water fraction within tumors as compared to normal tissues [6,7].

Computerized image analysis offers a means to increase the range of biologically relevant parameters available from an individual quantitative image. A commonly used method calculates an array of measures reflecting the distribution of image intensity values within a tumor region displayed as a histogram. Texture analysis (TA) represents an extension of this approach, with one widely used technique employing image filtration to highlight specified image features prior to histogram analysis [8]. This filtration-histogram approach to TA has attracted attention to assess intra-tumoral heterogeneity [9,10,11]. Intra-tumoral heterogeneity is related to tumor aggression, as reflected by numerous studies reporting an association between tumor texture and patient outcome (survival and/or treatment response) for a wide range of image modalities and tumor types [12,13,14,15,16,17,18,19,20,21].

The biological relevance of imaging assessments of intra-tumoral heterogeneity has been further highlighted by radiogenomic associations between imaging heterogeneity and gene mutation status recently reported for a number of tumor types [22]. Multi-functional/parametric imaging signatures derived from TA have shown associations with PBRM1 mutation status in clear cell renal cell carcinoma on CT [23], KRAS mutation status in colorectal cancer on PET/CT [24], KRAS and EGFR mutation status in non-small cell lung cancer on CT [25,26], IDH mutation status (including 1p19q genotyping) in glioma on MRI [27], and KIT exon 11 mutation in gastrointestinal stromal tumors on CT [28].

Colorectal cancer (CRC) is the third most common malignancy, with 1.7 million new cases and 860,000 deaths worldwide in 2018 [29]. Imaging plays a key role in the staging of patients with this disease so that the most appropriate therapeutic strategy can be selected. Contrast-enhanced computed tomography (CT) remains the mainstay investigation for staging CRC but there has been recent interest in the potential for PET/MRI to provide additional value by allowing better characterization of loco-regional lymph nodes and greater sensitivity to metastatic lesions, particularly in the liver [30,31]. However, PET/MRI is a costly procedure and it is uncertain whether the added benefits of PET/MRI will justify the additional costs if this modality is used for diagnosis alone. The ability to generate multiple quantitative images from a single examination represents a major advantage for PET/MRI over other imaging modalities. Each quantitative image provides a potential source for biomarkers, which could feasibly increase the clinical value and cost-effectiveness of PET/MRI in oncology [1].

The feasibility of applying TA to PET and MRI data acquired during PET/MRI has been reported [2,32,33], but none of these studies used TA to assess the heterogeneity of tumor fractional water content (FWC) as measured from Dixon sequences. Our study therefore aims to assess the capability of texture analysis of FWC images to generate additional biologically relevant information from routine PET/MRI acquisitions, with the potential to extend clinical application beyond simple diagnosis for patients with colorectal cancer.

## 2. Materials and Methods

### 2.1. Study Design

**Figure 1:**
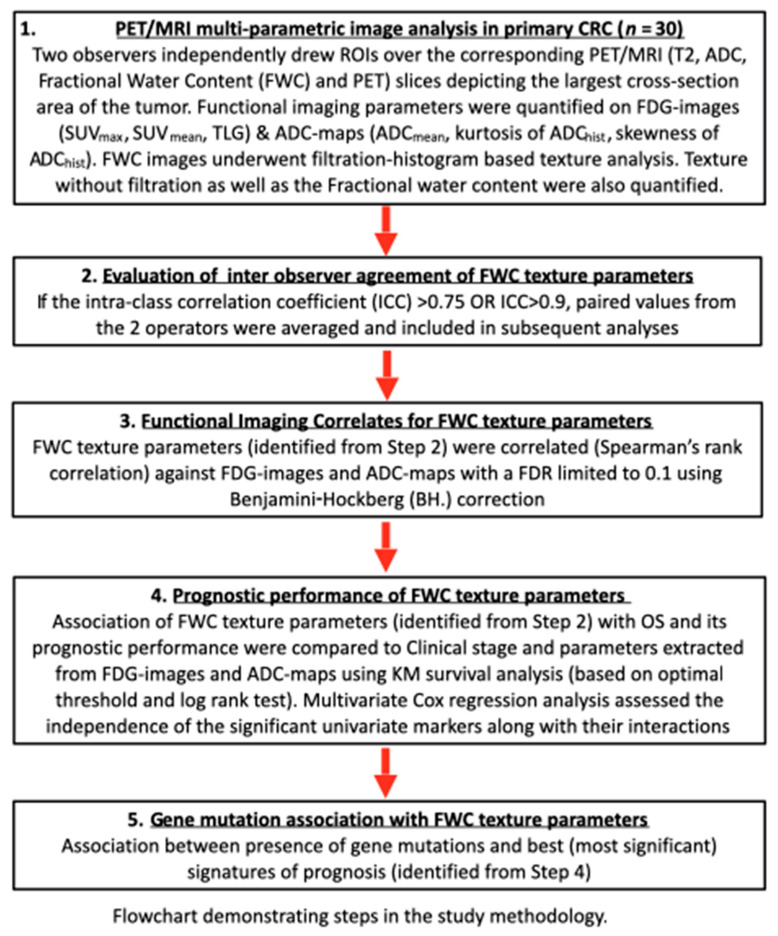
Outline of study design.

A prospective observational pilot study was performed. The study protocol was approved by the local Institutional Ethics Review Board. The study design comprised 3 components, as summarized in Figure 1: (1) an evaluation of inter-observer agreement of texture parameters, (2) identification of texture correlates for functional imaging parameters known to be related to tumor aggression, (3) an assessment of prognostic performance by analysis of Overall Survival (OS), and (4) association between presence of gene mutations and imaging signatures of prognosis.

### 2.2. Patients

Thirty consecutive newly diagnosed primary colorectal cancer (CRC) patients were recruited from February 2015 to May 2016 (20 males, 10 females; mean age 63.9 years; range 42–83 years). All patients gave informed consent prior to simultaneous FDG PET/MRI examination. OS was determined from clinical follow-up (median 25.4 months, range 5.0–31.6 months).

### 2.3. PET/MRI Image Acquisition

#### 2.3.1. PET

Following a 6-hour fast, patients received an intravenous injection of 250 MBq ^18^F-FDG ± 10%. After an uptake period of 60 min, all imaging was performed on an integrated PET/MR instrument (3T mMR Biograph, Siemens Healthcare, Erlangen, Germany). The PET emission scan was obtained over the same anatomical area. All acquisitions were carried out in 3-dimensional mode, consisting of an emission scan of 5 min/bed position. Reconstruction of PET data was carried out using ordered subset expectation maximization (OSEM) with 21 subsets and 3 iterations and a 5 mm full width half maximum (FWHM) Gaussian filter (FoV 359 × 359 mm, 2 mm slice thickness, 127 slices).

#### 2.3.2. MRI

During this time, a 3-dimensional (3D) T1 2-point Dixon sequence was performed with acquisition parameters as follows: axial, repetition time (TR) 4.13 ms, echo time (TE) 1–1.23 ms, TE 2–2.46 ms, FOV read 500 mm × FOV phase 390 mm, base resolution 320, voxel size 2.1 × 1.6 × 3.0 mm, 80 slices × 3 mm thickness, and 10% slice oversampling. Images interpolated. Breath hold time was 16 s.

A 2D DWI was also performed with acquisition parameters as follows: axial, TR 8400 ms, TE 89 ms, averages 4, FOV read 350 mm × FOC phase 274 mm, base resolution 184, voxel size 1.9 × 1.9 × 4 mm. SPAIR Fat Suppression, 32 slices × 4 mm slice thickness with a slice gap of 0.8 mm, and 3 × b values—0, 400, 800 s/mm^2^. An apparent diffusion coefficient (ADC) map was then calculated. Total scan time was 9 min 23 s.

An axial T2 half fourier single shot turbo spin echo (HASTE) was also performed to help localize the tumor but was not included in the analysis of this study.

### 2.4. Multi-Parametric Image Analysis

Quantitative image parameters were derived from attenuation-corrected FDG-PET data, ADC maps, and fractional water content (FWC) images (derived from water-only and fat-only images acquired as part of the 2-point Dixon sequence).

Two independent operators constructed tumor regions of interest (ROIs) enclosing the primary lesion from the PET/MR image displaying the largest cross-section area of the colorectal tumor. Each operator employed a standardized procedure so that ROIs were comparable in terms of anatomical location for all image data sets as follows. With reference to the T2 HASTE, the axial T2 slice with the largest cross-sectional diameter of tumor was selected. Multiple b value images were studied, and using this, an ROI was placed on the ADC map around the tumor site using Osirix MD software (v8.0.2-Pixmeo SARL, Geneva, Switzerland). The fat and water images corresponding to the ADC image slice and T2 image with the largest axial diameter were selected to generate the FWC image. The FWC image was created by dividing the individual pixel value from the water-only image by the sum of the individual and corresponding pixel values from water-only and fat-only images. Pixel size of the FWC image is 0.78 × 0.78 mm.

**Figure 2:**
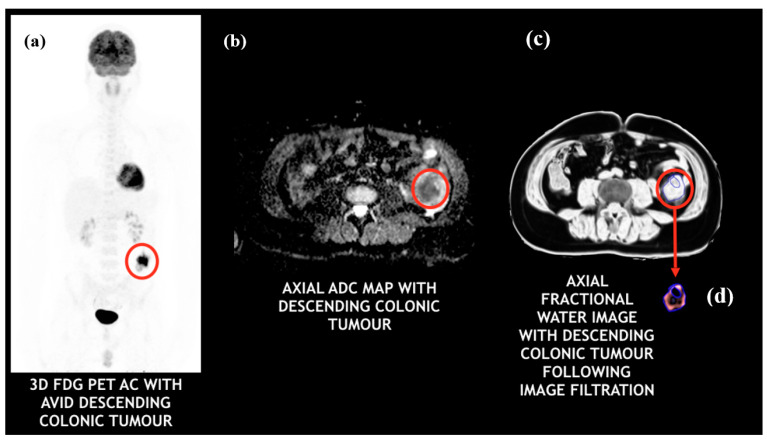
PET/MRI images of a colorectal cancer patient with a descending colonic tumor: (**a**) FDG-PET (tumor appears to be intensely avid); (**b**) ADC map (tumor appears to be highly cellular); (**c**) fractional water image (tumor appears to have very high water content); (**d**) fractional water texture map following coarse image filtration (bright/intense objects representing pockets of high water distribution within the tumor).

The PET emission study and MR were viewed independently and as co-registered studies using a commercial workstation (Horos v3.3.6—Horos is a free and open source code software-FOSS program that is distributed free of charge under the Lesser General Public License-LGPL at horosproject.org and sponsored by Nimble Co LLC d/b/a Purview in Annapolis, MD, USA, Figure 2). The tumor ROI on the PET image (comparable to the above MR analysis in slice location and ROI size) was obtained for each patient using an automated threshold method (42% of the maximum value was used as a default setting).

FDG uptake within the tumor was expressed as SUV_max_, SUV_mean_ and TLG. Tumor ADC_mean_ values were determined from ADC maps, as well as kurtosis and skewness values from ADC histograms. FWC images generated from the Dixon sequences underwent texture analysis using a commercially available proprietary software called TexRAD (Feedback Medical Ltd., Cambridge, UK, https://fbkmed.com/texrad-landing-2/, accessed on 23 April 2021). This software derives 36 texture parameters by applying a filtration-histogram technique in which the filtration step using a Laplacian of Gaussian filter to highlight image features of a specified size as determined by Spatial Scale Factor (SSF). A total of 5 SSF values were used (2, 3, 4, 5, and 6 mm, where SSF = 2 corresponded to fine texture scale, SSF = 3–5 corresponded to medium texture scale, and SSF = 6 corresponded to coarse texture scale). For each filter value, and for unfiltered data (SSF = 0), the following statistical and histogram-based parameters were derived: mean intensity, standard deviation (SD), entropy, mean of positive pixels (MPP), skewness, and kurtosis. Figure 2 provides an illustration of a FWC image and the filtration process. A detailed description of the above image filtration and quantification is described [34] and a computer modelling study has characterized the meaning of filtration-histogram-based texture features in terms of image features and how they relate to different components (object size, density, number) of heterogeneity [8].

### 2.5. Molecular Biology

Formalin fixed paraffin embedded tissue blocks (FFPE) were cut at a thickness of 10 µm followed by DNA extraction using the Qiagen QIAamp DNA FFPE tissue kit, according to manufacturer protocols [35]. Sequencing libraries were generated by loading 10 ng of genetic material into the Fluidigm Access Array IFC Chip with custom designed targeted primers for KRAS (exon 2, 3, and 4), NRAS (exon 2, 3, and 4), HRAS (exon 2 and 3), BRAF (exon 11, 12, and 15), PIK3CA (exon 2, 5, 8, 10, 14, 19, and 21), PTEN (exon 2, 4, 6, 7, 8, and 9) and APC (exon 15 and 16). Samples were barcoded and assessed using Agilent high sensitivity d1000 screen tape [36]. Samples were diluted to 100 pM and next-generation sequencing was undertaken using the Ion Torrent Personal Genome Machine, according to manufacturer protocols [37,38]. Data generated were analyzed on the Integrative Genomics Viewer (Broads Institute). Samples were compared to the human HG19 reference genome with a mutation frequency of 5% or above.

### 2.6. Statistical Analysis

All statistical analyses were performed using SPSS 25.0 (IBM Corp, released 2017, IBM SPSS Statistics for Mac, Armonk, NY, USA).

#### 2.6.1. Inter-Operator Agreement of Texture Parameters

The inter-operator agreement of FWC texture parameters was assessed by determining intra-class correlation coefficients (ICC) for each parameter. Paired values from the two operators were averaged for all parameters showing good (ICC > 0.75) or excellent agreement (ICC > 0.9) [39] and included in subsequent analyses.

#### 2.6.2. Functional Imaging Correlates for FWC Texture Parameters

Correlations were sought between each FWC parameter and the quantitative values derived from FDG images (SUV_max_, SUV_mean,_ TLG) and ADC maps (ADC_mean_, kurtosis, and skewness from ADC histograms). These functional imaging parameters are considered to relate to tumor aggression in CRC or other tumors. The strength of each correlation was determined using the Spearman rank correlation coefficient (r_s_). The false discovery rate was limited to 0.1 using the Benjamini–Hochberg (BH) correction procedure.

#### 2.6.3. Prognostic Performance of FWC Texture Parameters

FWC texture parameters significantly associated with OS were identified using Kaplan-Meier (KM) survival analysis and compared to the prognostic performance of quantitative values determined from the FDG images and ADC maps. The analyses were performed using optimal threshold values for separating good and poor prognostic groups. Differences between survival curves were evaluated using a non-parametric log rank test. A multivariate Cox regression analysis comprising of the best significant univariate markers along with their interactions was used to determine which parameters were independent predictors of survival (along with the hazard ratio (HR) and the 95% confidence interval (CI)).

#### 2.6.4. Gene Mutation Association with FWC Texture Parameters

For the FWC texture, FDG uptake and ADC metrics showing the most statistically significant associations with survival as identified above, a non-parametric Mann–Whitney test assessed the difference in the total number of gene mutations per patient in the good prognostic group from the poor prognostic group as identified by that imaging marker. In addition, a 2 × 2 contingency table assessed the presence or absence of specific gene mutations in the good and poor prognostic group as identified by the imaging marker and its significance was evaluated by Fisher’s exact test (2-tailed)

A *p*-value of less than 0.05 indicated a significant difference.

## 3. Results

Number of patients with clinical stages I, II, III, and IV were 2, 7, 9, and 12, respectively. Mean tumor fractional water content (FWC) was 0.88 (range: 0.16–0.98). Mean tumor ROI size was 1768 (range: 445–6233) pixels. The mean and range for all the FW imaging parameters used in the study are given in the Supplementary Materials (Table S1).

### 3.1. Inter-Operator Agreement of FWC Texture Parameters

ICC values for FWC texture values ranged between −0.15 and 0.98 (see Table S2 for details). Inter-observer agreement was good (ICC > 0.75) for 18 parameters (50%) and excellent (ICC > 0.9) for 14 (39%). Four FWC texture parameters demonstrating poor or moderate agreement (ICC < 0.75) were excluded from subsequent analyses: SD, kurtosis, skewness at SSF = 0, and skewness at SSF = 2.

### 3.2. Functional Imaging Correlates for FWC Texture Parameters

**Figure 3:**
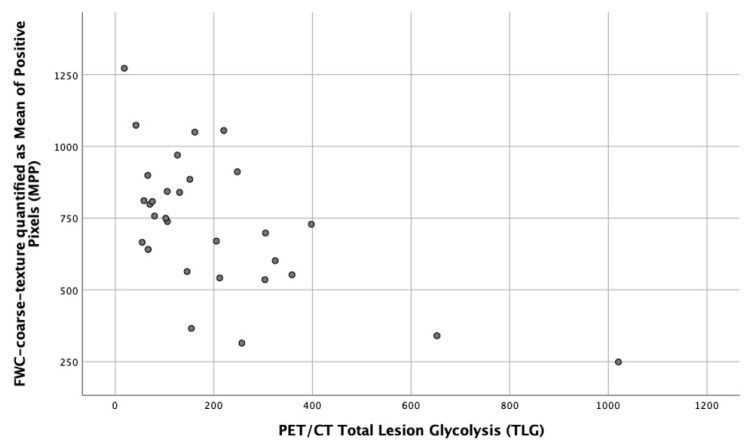
Scatter plot showing the correlation between fractional water texture expressed as MPP at coarse texture scale, SSF = 6 mm and TLG (r_s_ = −0.547, *p* = 0.002).

Eighteen texture parameters were found to be significant correlates for TLG (Table S3). The strongest correlation was for MPP at coarse texture scale, SSF = 6 mm (r_s_ = −0.547, *p* = 0.002, Figure 3). Four of five parameters with the strongest correlations (i.e., r_s_ values above 0.5 or below −0.5) were for medium or coarse texture features (SSF 5 or 6 mm). No unfiltered texture parameters (including the FWC measure) were shown to be significant correlates for TLG. No significant FWC texture (including the FWC measure) correlates were identified for SUV_max_ or SUV_mean_, nor for any ADC parameter.

### 3.3. Prognostic Performance of FWC Texture Parameters

Seven patients (23%) died during the follow-up period (median 25 months, range 5–32 months). Mean survival was 27 (95% confidence interval: 24–30) months.

The parameters significantly associated with survival included five derived using texture analysis of FWC images (Table 1).

**Figure 4:**
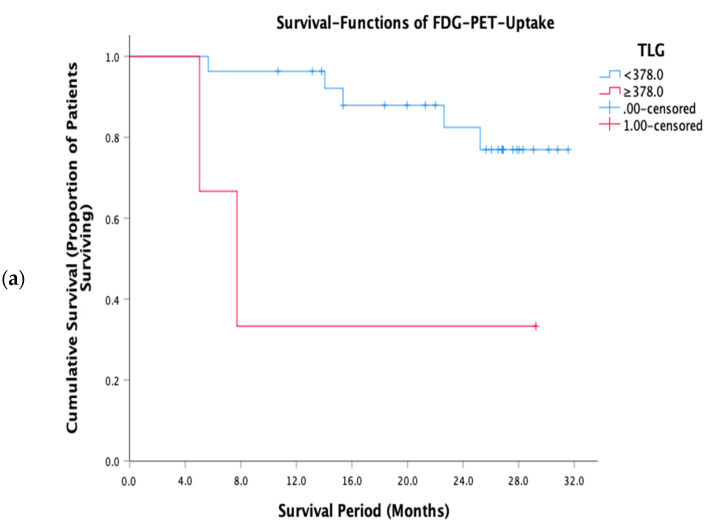

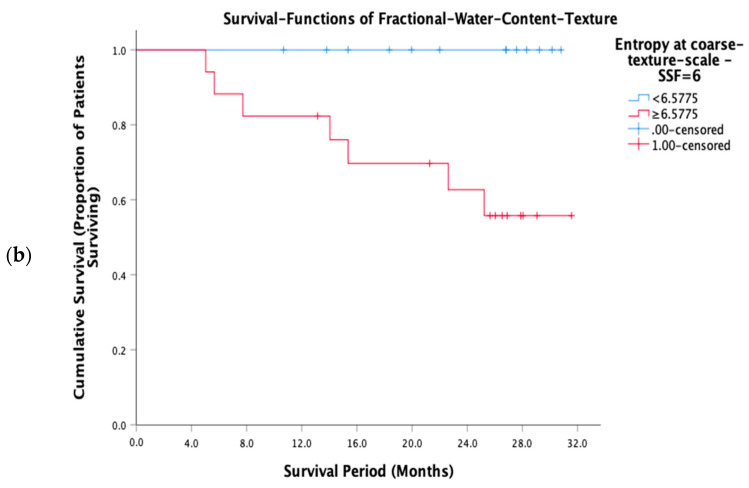
Kaplan-Meier survival curves for (**a**) FDG-PET uptake expressed as TLG and (**b**) FWC texture expressed as entropy at coarse texture scale, SSF = 6 mm.

FWC texture expressed as entropy was significantly associated with OS for all SSF values between 2 and 6 mm, being most significant for coarse texture scale, SSF = 6 mm (*p* = 0.017). In each case, there were no deaths in the good prognosis group. Two FDG uptake parameters (TLG and SUV_mean_) were significantly associated with survival, with TLG achieving greater statistical significance (*p* = 0.016 vs. 0.047). The skewness of the ADC maps was also significantly associated with survival (*p* = 0.023). In terms of clinical stage, as expected, patients with higher clinical stage (≥III) had a worse outcome compared to patients with lower clinical stage (<III), but not reaching statistical significance (*p* = 0.061). Good prognostic group as defined by lower clinical stage (<III) demonstrated zero mortality. Figure 4 compares the survival curves for FWC texture parameter entropy at coarse texture scale, SSF = 6 mm and FDG-PET uptak e quantified as TLG. Entropy at SSF = 6 was among the FWC texture correlates for TLG and the statistical significances of the two survival curves are similar. However, the value of TLG as a prognostic marker is constrained by the small proportion of patients falling within the poor prognostic group (3 of 30). For entropy SSF = 6, the sizes of the poor and good prognostic groups are more similar (17 and 13, respectively). Furthermore, the good prognostic group defined using entropy demonstrated zero mortality.

A multivariate Cox regression analysis comprising of the best FWC texture (entropy at SSF = 6), FDG uptake (TLG), and ADC (skewness of ADC maps) parameters demonstrated an interaction between FWC texture (entropy at SSF = 6) and FDG uptake (TLG) was the only best independent predictor of survival (HR = 44.7, 95% CI = 4.0–505.5, *p* = 0.002, Table 2).

### 3.4. Gene Mutation Association with FWC Texture Parameters

**Figure 5:**
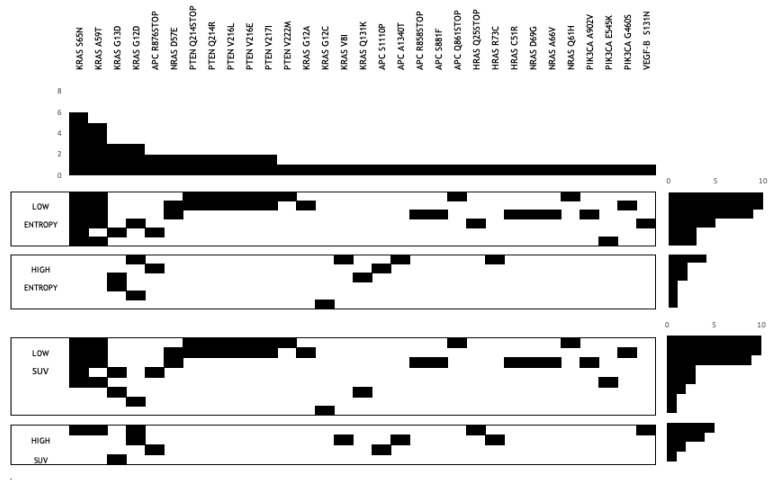
Gene map representing all gene mutation status available for the 12 patients divided into the good (low value of the imaging marker) and poor (high value of the imaging marker) prognostic groups as identified by FWC texture parameter (entropy at SSF = 6) and FDG uptake (SUV_mean_).

Gene mutation status was available for 12 of 30 patients. Amongst the prognostic imaging markers, FWC texture entropy at SSF = 6 had significantly higher number of gene mutations per patient in the good prognostic (low entropy) group (medians 7 vs. 1.5, *p* = 0.009). Specifically, KRAS S65 N and KRAS A59 T gene mutations were significantly higher in the good prognostic (low entropy) group as identified by FWC texture entropy at SSF = 6 (*p* = 0.002 and *p* = 0.015, respectively). Another gene mutation PIK3CA was significantly (moderately) higher in the good prognostic (low entropy) group as identified by FWC texture entropy at SSF = 2–5 (*p* = 0.045). Figure 5 shows a gene map representing all gene mutation status available for the 12 patients divided into the good (low value of the imaging marker) and poor (high value of the imaging marker) prognostic groups as identified by FWC texture parameter (entropy at SSF = 6) and FDG uptake (SUV_mean_).

## 4. Discussion

Authors should discuss the results and how they can be interpreted from the perspective of previous studies and of the working hypotheses. The findings and their implications should be discussed in the broadest context possible. Future research directions may also be highlighted.

Extending the use of PET/MRI beyond simple diagnosis has been identified as a means to add clinical value beyond PET/CT or MRI alone to enable this technology to become a cost-effective imaging modality in clinical practice [1]. In this pilot study, we demonstrate the potential for texture analysis of fractional water content images derived from Dixon sequence (also used for AC) to meet this requirement. We show that texture analysis of fractional water content (FWC) images benefits from good inter-observer agreement, generates biological relevant information related to TLG and gene mutation count, and is associated with OS for patients with primary colorectal cancer. The use of routinely acquired Dixon sequences (which could well be part of the AC images) facilitates incorporation of the technique into clinical workflows.

The authors have been unable to identify previous research reporting tumor FWC values derived using two-point Dixon sequences. Therefore, it is not possible to directly compare our results for inter-observer agreement to previous research. We have calculated tumor water content because of its greater relevance to tumor biology than fat content. Nevertheless, our method for calculating FWC is analogous to determination of signal fat fraction (FF) using two-point Dixon sequences, which is a parameter used clinically (FWC = 1 − FF). The ICC values for inter-observer agreement from our study are comparable to those reported for FF values derived from two Dixon images (i.e., 1 − water-fraction) in muscle [40,41].

FWC texture parameters reflect the distribution of water within the tumor tissue. The correlation we found between FWC texture and TLG points to the biological significance of tumor water distribution, as TLG reflects a combination of two important tumor characteristics, overall size and glycolytic activity. Coarse texture entropy was also an FWC texture parameter that correlated significantly with TLG in our study (r_s_ = 0.498, *p* = 0.005, Table S3).

Seven of the ten strongest FWC texture correlates for TLG comprised negative associations with either mean intensity values or the mean of positive pixels. These texture parameters increase in value depending on the number and intensity of bright objects highlighted by the filtration step [8]. This finding suggests that tumors containing more focal areas with high water content tend to be smaller and/or less glycolytic. Several possible explanations can be proposed for an inverse relationship with glycolysis. Areas with higher fractional water content could represent areas with greater cellular density but with a high proportion of non-glycolytic cells. Alternatively, a high local water content could reflect more extracellular fluid or regions of necrosis. Further work will be required to determine the biological correlates for FWC texture parameters.

Five FWC texture parameters were significantly associated with OS, of which four were also identified as correlates for TLG. We also found TLG to be the metabolic parameter most significantly associated with survival, consistent with a previous study of colorectal cancer patients prior to treatment [42]. Our threshold value for TLG in determining prognostic groups (≥378 indicating poor prognosis), and the proportion of patients above the threshold (10%) were also similar to that study (342 and 13%, respectively). In both cases, the mismatch in the sizes of the prognostic groups is a constraint on the usefulness of TLG as a prognostic marker. In comparison, the sizes of good and poor prognostic groups were much more even for FWC texture parameters, while the statistical significance for the association between FWC texture expressed as coarse entropy and OS was close to that found for TLG. FWC texture also benefited from identifying a good prognosis group with zero mortality.

Genetic analysis demonstrated a high tumor gene mutation count in patients with good prognosis as measured by low FWC texture entropy. Previous reports have hypothesized that a higher number of gene mutations results in a greater number of tumor specific neo-antigens [43]. This, in turn, stimulates an infiltration of T-lymphocytes and thus an up-regulation of immune checkpoints. An alternative hypothesis for good prognosis is that cell viability decreases as genomic instability increases, as reflected by a greater number of gene mutations [44]. Significant mutations seen in the genetic analysis included KRAS A59T and S65N. KRAS is a well-documented activating driver mutation associated with CRC. Missense mutations in codons 12 and 13 on exon 2 are reported in over 75% of CRC cases and linked with no response to anti-EGFR therapy [45]. KRAS S65N mutation is not reported in the literature and appears to be a novel mutation associated with CRC. Interestingly, case studies have found KRAS A59T positive CRC respond to anti-EGFR therapy, which is contrary to current treatment guidelines for KRAS positive tumors [45].

Texture analysis using the filtration-histogram approach has also been applied to CT images of primary tumors, including the low-dose CT used for attenuation correction in PET/CT [16]. Correlations between CT texture and tumor metabolic parameters have been demonstrated for non-small lung cancer (NSCLC) and esophageal cancer [46,47], and between CT texture and OS in colorectal cancer [15]. In all of these examples, CT texture quantified as entropy was among the significant parameters, corresponding to the prominence of entropy values in our study. Although the limitations of entropy as a surrogate for tumor heterogeneity have been highlighted [48], this parameter may represent a means of quantifying tumor heterogeneity regardless of imaging technique. Nevertheless, the application of texture analysis to the routinely acquired Dixon sequences (which could well be used for AC increasing the utility of existing images) part of PET/MRI has advantages over a comparable use in PET/CT, including reduced radiation exposure and the ability to combine texture analysis with a wider range of other quantitative imaging techniques. The importance of filtration-histogram approach to texture analysis is further highlighted by the fact that no unfiltered texture parameters (including the fractional water content measure, although generally higher in colorectal tumors) showed any significant association with functional imaging parameters, patient prognosis and gene mutation profile.

A further notable finding in our study is that histogram analysis of tumor ADC values can also potentially provide prognostic information for patients with colorectal cancer. The capacity for kurtosis and/or skewness values of ADC histograms to distinguish benign and malignant lesions, and to predict or assess treatment response has been shown for a range of tumor types [49]. In rectal cancer, the skewness of the ADC histogram was found to be related to tumor stage and the presence of extramural invasion [50]. However, inclusion of DWI within PET/MRI protocols creates economic challenges for this modality. The time required for DWI is much longer than that needed for Dixon sequences, extending the image acquisition time beyond that required for acquisition of PET data. In these circumstances, the PET/MRI system is effectively being used for MRI alone for significant periods. This mode of utilization is economically undesirable given the high cost of these devices. In addition, standard whole-body DWI acquisitions used in PET/MRI are subject to distortion artefacts, which can lead to mis-registration between PET and MRI datasets. A recent PET/MRI study of 20 oncological patients reported 19 distortion artefacts when using standard DWI sequences [51]. The frequency of artefacts increased when a simultaneous multi-slice technique was used to reduce acquisition time. Anatomical distortion during DWI can be reduced by the use of multi-shot echo planar imaging, but these techniques require even longer image acquisitions, further increasing the economic challenges. Furthermore, biomarkers derived from Dixon sequences part of MR-only acquisition have been shown to benefit from superior reproducibility as compared to ADC values [52], providing insights in pathophysiology [53] and early response to treatment [54]. In addition to the benefits of superior reproducibility of Dixon sequences, the use of filtration-histogram-based texture analysis has demonstrated less variance (increased robustness) to image acquisition parameter changes [55,56].

This study is limited by the relatively small number of patients, commensurate with an initial, pilot feasibility study, whereby we could not apply a power analysis to determine an appropriate size of the study population. However, we undertook an inter-operator agreement between the two readers for the FWC texture quantification and only the parameters that showed good or excellent ICC were included for subsequent analysis. To address the multiple comparison issue arising in the process of identifying a functional imaging correlate for FWC texture parameters, the Benjamini–Hochberg correlation was applied to reduce the false discovery rate. It is worth noting that the tumor ROI contour was drawn on a single axial slice. Although the multi-slice or volume delineation would be a better representation of the whole tumor, such a methodology is time-consuming and therefore not practical in a clinical setting. Furthermore, comparable results in texture-based heterogeneity assessment have been reported between cross-sectional area and whole volume analysis in colorectal cancer on CT [57] and gliomas on MRI [27]. Finally, the use of optimized threshold values for survival analysis may have over-estimated the prognostic ability, not only for FWC texture but also tumor metabolic and ADC parameters. The potential prognostic value of FWC texture needs to be confirmed in larger studies with separate cohorts for derivation of threshold and validation of prognostic performance. Our study methodology, although keeping in mind the feasibility nature, has been described in detail above, which more or less adheres to the REMARK guidelines/checklist aimed at improving the reporting of these types of outcome studies [58]. It will be recommended to follow the REMARK guidelines/checklist in future studies validating our feasibility results in a larger prospective population.

Two general applications beyond simple diagnosis that have been identified as having the potential to render PET/MRI cost-effective in clinical practice are risk-adapted post-operative surveillance and selection of patients for targeted therapy [1]. Both scenarios are highly relevant to patients with colorectal cancer. Most patients with operable colorectal cancer undergo post-operative surveillance with a view to early detection of treatable tumor recurrence. The efficacy of surveillance can be increased, and costs reduced by de-escalating follow-up in patients with a good prognosis [59]. There has also been recent interest in the use of immune checkpoint inhibitor therapy for colorectal cancer in the neoadjuvant setting [60]. PET/MRI markers could potentially identify those patients most likely to benefit from these agents and/or those who may need additional adjuvant chemotherapy. The association we found between prognostic group as determined by FWC texture and gene mutation count is particularly germane in this context, given the recognized importance of tumor mutation burden in determining response to immune checkpoint inhibitors. Although further research specific to these scenarios is required, the prognostic ability shown in our study suggests that FWC texture analysis may able to unlock these clinical applications for PET/MRI.

## 5. Conclusions

The use of imaging-based texture analysis as a marker of tumor heterogeneity for determining tumor prognosis, characterization and assessing response to therapy is gaining momentum as a clinical tool [10]. Texture analysis of FWC images derived from routine PET/MRI Dixon acquisitions (also part of the AC images) shows good inter-operator agreement, generates biological relevant information related to TLG and gene mutation count, and provides prognostic information that can potentially unlock new clinical applications for patients with colorectal cancer.

## Figures and Tables

**Table 1:** FWC texture and functional imaging parameters significantly associated with overall survival.

Parameter	Threshold (Direction Indicates Poor Prognosis)	Patients above/below Threshold	*p*-Value
Texture			
Entropy SSF = 2	≥6.19	19/11	0.033
Entropy SSF = 3	≥6.44	18/12	0.024
Entropy SSF = 4	≥6.52	18/12	0.024
Entropy SSF = 5	≥6.53	18/12	0.024
Entropy SSF = 6	≥6.58	17/13	0.017
FDG uptake			
SUVmean	≥10.1	9/21	0.047
TLG	≥378	3/27	0.016
ADC maps			
Skewness	≥0.61	3/27	0.023

**Table 2:** Summary of multivariate Cox regression analysis model comprising of the most significant univariate FWC texture, FDG uptake, and ADC parameters.

**Parameter Included in the Model**	**HR**	**95% CI**	***p*-Value**
Entropy SSF = 6 * TLG	44.7	4.0–505.5	0.002
**Parameters Not Included in the Model**	**Score**		***p*-Value**
Entropy SSF = 6	4.1		0.042
TLG	0.3		0.613
Skewness of ADC maps	0.03		0.867
TLG * Skewness of ADC maps	1.7		0.190
Entropy SSF = 6 * Skewness of ADC maps	0.03		0.867

* indicates the interaction between the covariates.

## Data Availability

Data are available on request due to restrictions concerning privacy and ethical. The data presented in this study may be available on request from the corresponding author pending internal considerations and approvals. The data are not publicly available due to above reasons.

## Supplementary Materials

The following are available online at https://www.mdpi.com/article/10.3390/cancers13112715/s1, Table S1: Mean and range of all the imaging parameters employed in the study, Table S2: Intra-class correlation (ICC) values characterizing the inter-observer agreement for each texture parameter, Table S3: FW texture correlates for TLG (false discovery rate limited to 0.1).
