# Supplementary material for: Texture Analysis of Fractional Water Content Images Acquired during PET/MRI: Initial Evidence for an Association with Total Lesion Glycolysis, Survival and Gene Mutation Profile in Primary Colorectal Cancer
Source: Cancers (Basel). 2021 May 31;13(11):2715. doi: 10.3390/cancers13112715 (PMC8199380; doi:10.3390/cancers13112715)
Supplement: Supplementary file 1 [file cancers-13-02715-s001.zip › cancers-1217293-supplementary.pdf]

Supplementary Material: Texture Analysis of Fractional Water Content Images Acquired During PET/MRI: Initial Evidence for an Association with Total Lesion Glycolysis, Survival and Gene Mutation Profile in Primary Colorectal Cancer

Balaji Ganeshan, Kenneth Miles, Asim Afaq, Shonit Punwani, Manuel Rodriguez, Simon Wan, Darren Walls, Luke Hoy, Saif Khan, Raymond Endozo, Robert Shortman, John Hoath, Aman Bhargava Matthew Hanson, Daren Francis, Tan Arulampalam, Sanjay Dindyal, Shih-Hsin Chen, Tony Ng and Ashley Groves

Table S1. Mean and range of all the imaging parameters employed in the study.

Tumor Characteristic	Mean	Range
Fractional water content	0.88	0.16–0.98
MRTA of FW (without-filtration, SSF = 0)		
SD	82.79	16.97–146.35
Entropy	5.12	3.51–5.79
MPP	0.88	0.16–0.98
Skewness	−1.57	−2.41–1.61
Kurtosis	3.66	−6.79–10.95
MRTA of FW (fine-scale, SSF = 2)		
Mean	144.18	−65.21–399.56
SD	257.96	108.75–359.73
Entropy	6.23	5.27–6.76
MPP	246.80	78.06–439.36
Skewness	0.96	0.12–2.22
Kurtosis	1.88	−0.17–11.41
MRTA of FW (medium-scale, SSF = 3)		
Mean	276.68	−89.23–705.07
SD	341.82	161.74–499.62
Entropy	6.42	5.55–6.85
MPP	379.83	123.35–773.96
Skewness	0.68	−0.06–2.27
Kurtosis	0.68	−0.83–8.99
MRTA of FW (medium-scale, SSF = 4)		
Mean	413.91	−98.89–952.59
SD	391.55	208.89–571.80
Entropy	6.53	5.73–7.02

MPP	505.52	172.55–1034.12
Skewness	0.43	−0.19–2.05
Kurtosis	0.08	−1.21–5.37
MRTA of FW (medium-scale, SSF = 5)		
Mean	544.79	−101.12–1132.39
SD	414.49	245.39–561.21
Entropy	6.58	5.87–7.16
MPP	620.92	212.76–1180.35
Skewness	0.23	−0.36–1.73
Kurtosis	−0.16	−1.24–2.98
MRTA of FW (coarse-scale, SSF = 6)		
Mean	665.06	−99.73–1257.67
SD	421.61	271.27–575.13
Entropy	6.61	5.93–7.21
MPP	730.95	248.84–1271.93
Skewness	0.07	−0.54–1.48
Kurtosis	−0.20	−1.34–1.64
FDG PET uptake		
SUV_{max}	16.46	8.42–52.11
SUV_{mean}	9.63	4.65–32.12
TLG	207.09	18.22–1020.61
ADC quantification		
ADC_{mean} from ADCmaps	981	122–1310
Skewness of ADC histogram	0.22	−0.80–0.89
Kurtosis of ADC histogram	0.16	−0.74–1.93

Table S2. Intra-class correlation (ICC) values characterizing the inter-observer agreement for each texture parameter.

Texture-scale, SSF	Mean	SD	Entropy	MPP	Skewness	Kurtosis
Without-filtration, SSF = 0	0.980	0.402	0.832	0.980	0.623	−0.152
Fine, SSF = 2	0.866	0.870	0.878	0.878	0.685	0.910
Medium, SSF = 3	0.899	0.901	0.879	0.929	0.814	0.889
Medium, SSF = 4	0.916	0.905	0.881	0.953	0.838	0.913
Medium, SSF = 5	0.927	0.871	0.871	0.956	0.875	0.901
Coarse, SSF = 6	0.931	0.805	0.879	0.951	0.873	0.779

Table S3. FW texture correlates for TLG (false discovery rate limited to 0.1).

**Texture-Parameter**	**SSF (mm)**	**r_s**	***p*-value**
MPP	6	−0.547	0.002
Mean intensity	6	−0.541	0.002
Mean intensity	5	−0.530	0.003
Kurtosis	6	0.520	0.003
MPP	2	−0.503	0.005
Entropy	6	0.498	0.005
Mean intensity	4	−0.487	0.006
Mean intensity	3	−0.485	0.007
MPP	4	−0.479	0.007
Entropy	5	0.480	0.007
MPP	5	−0.478	0.008
MPP	3	−0.443	0.014
Mean intensity	2	−0.433	0.017
Entropy	4	0.394	0.031
Kurtosis	2	0.390	0.033
Entropy	3	0.385	0.036
Kurtosis	5	0.382	0.037
Kurtosis	3	0.375	0.041
